# Cognitive Control and Prefrontal Neural Efficiency in Experienced and Novice E-Gamers

**DOI:** 10.3390/brainsci15060568

**Published:** 2025-05-25

**Authors:** Jan Watson, Adrian Curtin, Yigit Topoglu, Rajneesh Suri, Hasan Ayaz

**Affiliations:** 1 School of Biomedical Engineering, Science and Health Systems, Drexel University, Philadelphia, PA 19104, USA; 2 Lebow College of Business, Drexel University, Philadelphia, PA 19104, USA; 3 Drexel Solutions Institute, Drexel University, Philadelphia, PA 19104, USA; 4 Department of Psychological and Brain Sciences, College of Arts and Sciences, Drexel University, Philadelphia, PA 19104, USA; 5 A.J. Drexel Autism Institute, Drexel University, Philadelphia, PA 19104, USA; 6 Center for Injury Research and Prevention, Children’s Hospital of Philadelphia, Philadelphia, PA 19104, USA

**Keywords:** first-person shooter (FPS), cognitive control, neuroergonomics, fNIRS, prefrontal cortex, digit–symbol substitution task (DSST), dual visual search task (DVST), Stroop task

## Abstract

**Background:** Cognitive control (CC) underpins our ability to maintain task focus, update goals, and flexibly shift between strategies, and it is closely tied to prefrontal cortex (PFC) activity. Electronic gaming (e-gaming), such as the first-person shooter (FPS) genre, is a specialized domain that demands refined CC skills developed over years of practice. Although previous research has demonstrated that PFC hemodynamic activity can effectively evaluate CC in several skilled domains, the impact of prolonged FPS experience on CC and its underlying neural correlates remains unclear. **Objectives:** In this study, we examined differences in both behavioral performance and PFC hemodynamic responses between 70 novices and 50 experienced FPS gamers (n=120). **Methods:** We targeted three core CC subdomains—updating, shifting, and inhibition—by employing the Digit–Symbol Substitution Task, Dual Visual Search Task, and Stroop Task, respectively. Functional near-infrared spectroscopy (fNIRS)-based PFC activity was recorded as participants engaged in each task. **Results:** Experienced gamers showed higher levels of prefrontal neural efficiency for updating and shifting, but minimal differences for inhibitory control. **Conclusions:** These findings inform neuroergonomic approaches to performance evaluation and may be generalized to other complex, real-world environments that rely on extensive training for skill acquisition.

## 1. Introduction

Cognitive control (CC) encompasses a suite of executive functions essential for goal-directed behavior, flexible attention, and adaptive problem-solving. These functions critically depend on the prefrontal cortex (PFC) and its associated networks [1,2], which regulate the balance between deliberate and controlled, versus more habitual, automated processing. The PFC, in particular, is responsible for facilitating adaptive behavior with rapid decision making and response control in dynamic environments by orchestrating cognitive control functions, including attentional focus, strategic flexibility, and inhibitory control [2,3].

Within the broader CC framework, three subdomains have been extensively examined in cognitive neuroscience: (i) information updating (“updating”), involving the continuous monitoring and manipulation of relevant information in working memory; (ii) mental set-shifting (“shifting”), enabling rapid adaptation to changing rules or task demands; and (iii) inhibition of prepotent responses (“inhibition”), necessary for suppressing dominant yet inappropriate actions [4]. Each subdomain is typically probed by distinct cognitive tasks that elicit characteristic PFC activation patterns. For example, the Stroop task reliably recruits the dorsolateral PFC when participants override the habitual inclination to read words rather than name colors [5,6]. Shifting is commonly assessed with paradigms that require switching between task sets [7], while both the N-back and the Digit–Symbol Substitution Task (DSST) are known to engage lateral anterior PFC regions that subserve working memory and attentional control [8,9,10].

First-person shooter (FPS) games represent a unique segment of the electronic gaming (e-gaming) space, demanding complex and rapid decision making that appears to draw heavily on all three CC subdomains [11,12,13,14]. From a first-person vantage, players must rapidly identify moving targets, detect threats, and continually shift attention among multiple objectives and environmental cues. This interactive environment necessitates continual updating of one’s internal representation of the game state, integrating new information as it emerges. At the same time, impulsive actions may carry fewer immediate consequences, potentially diminishing the need for strong inhibitory control. Indeed, previous work has suggested that prolonged exposure to FPS gaming does not show improvement in inhibitory responses [11,13,14], possibly explaining why improvements in task probing inhibition (e.g., the Stroop task) are not consistently observed among experienced FPS players.

Despite growing interest in how gameplay experience shapes CC, neuroimaging work has primarily focused on brain changes during or directly following gameplay [15,16]. Although numerous studies have addressed how FPS experience can influence behavioral performance on CC tasks [7,11,12,13], few have examined its long-term neural correlates [17,18]. This leaves important questions open about whether (and how) prolonged FPS exposure might reshape the neurophysiological underpinnings of CC, especially within the PFC—the primary locus of executive function [19,20].

To bridge this gap, the present study combined three tasks known to regulate the CC subdomains of updating (DSST), shifting (DVST), and inhibition (Stroop) with functional near-infrared spectroscopy (fNIRS) to capture PFC hemodynamic responses in novice versus experienced FPS gamers. By isolating each subcomponent of CC, our design aimed to clarify the extent to which years of FPS gaming alter both performance and PFC engagement across the three CC domains. The results of this investigation will not only shed light on the neurocognitive mechanisms of FPS expertise but also inform wearable neuroimaging applications in other complex real-world contexts where specialized training is pivotal.

## 2. Materials and Methods

### 2.1. Participants

A total of 120 participants (30 females, mean age = 21.7 years, SD = ±3.8 years) volunteered for the study and received monetary compensation for their time. All confirmed that they met the eligibility requirements of being right-handed with vision correctable to 20/20, did not have a history of brain injury or psychological disorder, and were not on medication affecting brain activity. Prior to the study, all participants signed consent forms approved by the Institutional Review board of Drexel University (Philadelphia, PA, USA). All participants had previous e-game experience and were divided into *novice* and *experienced* groups based on a questionnaire given prior to the experiment to assess e-gaming background.

Participants were asked to report their gameplay frequency as well as their length of experience with FPS gaming. If participants reported FPS genre gameplay experience as part of their profile (more than 12 months), participants were also asked to report the average hours per week that they played FPS games. If they reported FPS gameplay experience equal to or greater than 10 h per week, they were admitted to the study and classified as an experienced FPS gamer. If they reported less than 10 h per week of gameplay they were classified as novice FPS gamers. If no FPS gameplay was reported, participants were admitted to the novice group. For all participants admitted to the study, the median value of reported weekly gameplay was 10 h. This value corresponds to the average reported gameplay of US adolescents [21] and aligns with the demarcating value specified by previous FPS gaming behavioral research [7], showing that 10 or more hours of weekly action videogame play improved performance in a behavioral task. Qualifying FPS games included titles such as Overwatch, Call of Duty, Counter Strike, Halo, Half-Life, Medal of Honor, Fortnite, and Rainbow Six.

### 2.2. Experimental Procedure

**Figure 1:**
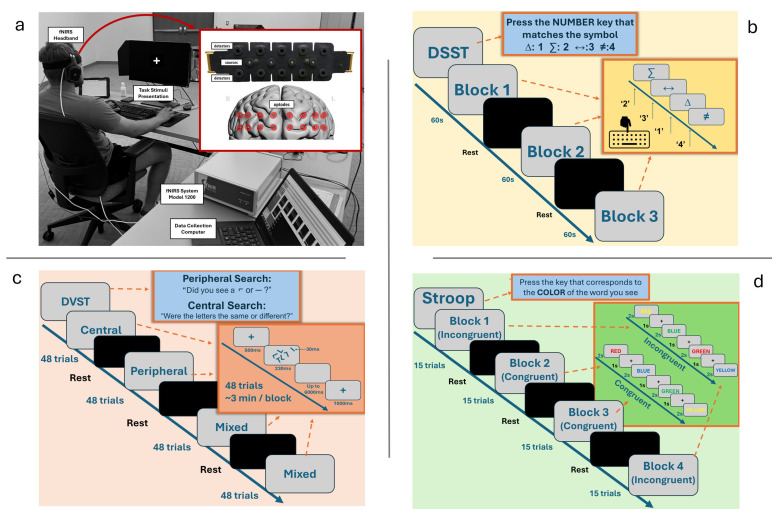
Experiment set-up and schematic description of tasks. (**a**) Participant sitting in front of task stimuli presentation with fNIRS flat sensor pad on forehead and respective measurement areas represented on the prefrontal cortex. (**b**) DSST representative instructions and flow. (**c**) DVST representative instructions and flow. (**d**) The Stroop Task outline with examples of both congruent and incongruent trials included.

We designed a discrete laboratory cognitive task battery to assess the CC subdomains of shifting, updating, and inhibition using discrete laboratory tasks that were previously demonstrated to evoke responses in the PFC [3]. Participants performed this CC task battery over a thirty-minute session while seated 18 inches from a 24-in LED monitor. All participants were fitted with a 16-optode continuous wave fNIRS system Model 1200 (fNIR Devices, LLC., Potomac, MD, USA) positioned on the forehead to measure prefrontal cortex hemodynamic activity. After device set-up, participants completed a Digit–Symbol Substitution Task (DSST), a Dual Visual Search Task (DVST), and Stroop Task (Figure 1).

#### 2.2.1. Digit–Symbol Substitution Task

Visual monitoring and speed of processing are fundamental characteristics of the FPS skillset as gameplay requires not only precise sensory–motor coordination but also a constant updating of goals and targets across the player’s visual field [22,23]. In three separate 1 min blocks of probe presentation (trials lasting a maximum duration of 2 s each), a symbol probe was presented visually at the center of the screen. Participants were required to press the number on their keyboard that corresponded to the correct symbol–digit pairing on a key at the top of their screen as quickly and as accurately as possible. Probe presentation and digit–symbol pairings were pseudorandomized across trials. Accuracy and reaction times were recorded, and participants received scores based on their number of correct responses per block.

#### 2.2.2. Dual Visual Search Task

FPS players often detect and identify threats in the periphery, while being simultaneously engaged in central search tasks. These demands require the ability to flexibly shift between cognitive strategies. In our laboratory analog, participants performed two simultaneous search tasks that required discrimination and identification in central and peripheral areas of the visual field [24]. When performing both searches simultaneously, a cost to attentional resources is incurred due to switching from one search strategy to the other. For the central search condition, participants were required to report whether five randomly rotated letters were identical. During the peripheral search condition, participants had to locate and identify a briefly presented target (either “¬” or “-”) that could appear anywhere and with any rotation in a circular locus in the periphery (peripheral search condition). In the dual-search condition, participants performed the peripheral and central searches and were randomly asked to respond to either the central or peripheral search queries. By presenting both search conditions separately as well as within a dual-task context, both baseline performance and task-switching costs could be evaluated. Accuracy and RTs were recorded.

In four separate task blocks, the participants completed three task conditions. These included one block for each single-search condition: peripheral search only, where participants were asked which symbol they saw (¬ or ), and central search only, where participants were asked if they saw identical or different letters. Additionally, two blocks of dual-search tasks were performed, where both central search and peripheral search question trials were evenly distributed in each block. Each block consisted of 48 trials. The order of the individual trials was pseudorandomized, and the order of the four task blocks was counterbalanced across participants. Each of the tasks was carried out as follows.

*Peripheral Search Alone:* Participants were required to fixate at the center of the screen and to locate and identify the peripheral stimulus while ignoring the stimuli at the center. Because the position of the peripheral stimulus was chosen pseudo-randomly, participants could not improve their performance by fixating on a location other than the center. They did, however, know that the target would appear somewhere on the circumference of an imaginary circle in the periphery. Participants responded as quickly as possible while minimizing errors, pressing “7” if they identified a “¬” or “9” if they identified a “-”, depending on the symbol presented.

*Central Search Alone:* Participants were required to fixate at the center of the screen and to identify whether the group of five letters that appeared were all identical or different while ignoring the peripheral stimulus. The participants pressed the “7” key if the five central letters were identical, and the “9” if any letter was different.

*Dual-Search (Mixed):* Both search tasks were performed simultaneously. After the stimulus disappeared, a cue was presented that instructed participants to produce a response for either the central search or the peripheral search (equal probabilities across trials). Participants were expected to maintain performance in both conditions without prioritizing one task condition over another.

#### 2.2.3. Stroop Task

The Stroop paradigm examines an individual’s ability to shift their cognitive set in the presence of distraction and to suppress irrelevant information and maintain focus on a given task as a measure of cognitive inhibition and control [25]. Participants were asked to look at words (i.e., red, blue, yellow, green) in two different conditions: *congruent*, where the word was displayed in the color font that its name denoted (e.g., “yellow” displayed in yellow font), and *incongruent*, where the word was displayed in a different color font than its name (e.g., “yellow” displayed in red font). The task consisted of four blocks—2 congruent (15 trials per block) and 2 incongruent (15 trials per block)—with presentation pseudorandomized across participants to prevent an order effect. Stimuli presentation trials were two seconds in duration with one-second interstimulus rest intervals and thirty-second rest intervals between blocks. Accuracy and reaction times were recorded.

### 2.3. fNIRS Neuroimaging

#### 2.3.1. Data Collection

Hemodynamic response in the prefrontal cortex was measured using a continuous wave fNIRS system (fNIR Imager Model 1200; fNIR Devices, LLC., Potomac, MD, USA) first described by [26] and developed at Drexel University [27,28]. Light intensity at two near-infrared wavelengths of 730 and 850 nm and one additional ambient light channel from 16 optodes each were recorded at 2 Hz using COBI studio software (version 1.5.0.55) [28]. The fNIRS headband contains 4 light-emitting diodes (LEDs) and 10 photodetectors for a total of 16 optodes (measurement areas). The sensor was positioned based on the anatomical landmarks, as previously described [28], by aligning the center of the sensor to the midline of the forehead, with the bottom of the sensor touching the participants eyebrow so that the centroid point of the sensor was approximately at the prefrontal center (Fpz, where Fp refers to the frontopolar area of the prefrontal region, and Z as the central region), according to the 10–20 international coordinate system. Approximate anatomical mapping between fNIRS headband optodes and cortical regions is described in [29]. Baseline light intensity measurements were taken immediately preceding the start of the experiment. Time synchronization markers were sent from the stimulus presentation computer to the fNIRS acquisition computer for the registration of task responses, blocks, and trials for the temporal alignment of recorded data for all subjects.

#### 2.3.2. Preprocessing

Raw light intensity data were pre-processed using a low-pass finite impulse response (FIR) filter, with a filter order of 20 and a cut-off frequency of 0.1 Hz [30]. The fluctuations from motion artifacts in the filtered raw signal and hemoglobin concentration changes were inspected both visually and also by using the automated sliding motion artifact rejection (SMAR) algorithm [31], which uses a coefficient-of-variation-based statistical filtering to assess signal quality and reject problematic channels with poor contact or saturated raw light intensity. Time-synchronized blocks of fNIRS pre-processed light intensity signals for all task periods were processed using the modified Beer–Lambert law [32] and baseline-corrected to calculate oxygenated hemoglobin (HbO) changes.

#### 2.3.3. Analysis

Linear mixed-effects models (LMMs) were used for statistical analysis to account for intersubject variability and repeated measures [33]. Separate models were constructed to identify the impact of the fixed effects of interest on dependent measures of task response variables and respective biomarkers. LMMs incorporate random effects for individual participants while capturing variability between groups of participants and are flexible in handling missing data [34]. They do not require the same number of measurements across participants, which applies to fNIRS studies in which data may be missing due to technical issues or participant non-compliance. Finally, LMMs have been frequently applied in fNIRS studies, including those assessing cognitive processing [35,36].

For each task, model functionality was specified as follows: Outcome~Group+Condition+Group:Condition+(1|Subject). Where ‘Outcome’ represents the dependent variable (e.g., response time, accuracy, HbO, neural efficiency), ‘Group’ represents the experience level (novice vs. experienced), and ‘Condition’ represents the task-specific conditions. Random intercepts were included to account for individual differences.

Here, the statistical significance in model fixed effects was assessed using the Satterthwaite approximation for degrees of freedom. Post hoc tests were conducted using Tukey contrasts adjusted for family-wise error rates using the Bonferroni correction. A criterion of α = 0.05 was designated as a threshold for statistical significance. For fNIRS analysis, false discovery rate (FDR) correction was applied to LMM results to correct for multiple family-wise testing across the entire list of optodes (with *q* = 0.05) [37]. Bonferroni correction was applied during the post hoc analysis of individual contrast comparisons within an optode that survived the previous FDR correction [38].

The process of skill acquisition and training can be viewed as the acquisition of knowledge and the formulation of strategies that attempt to reduce relative cognitive demands and increase overall mental efficiency, thereby allowing for better performance. The demands of metabolically expensive neuronal activity involved in the maintenance of active cognitive resources imply that changes in mental efficiency are coupled with changes in neural efficiency (NE) [39]. In this study, neural efficiency analysis was conducted as a way of providing a consolidated metric of behavioral performance which relates mental effort to outcome [39,40], and it was calculated using the following formula: $\mathrm{NE}=\left[z\left(\mathrm{Performance}\right)-z\left(\mathrm{Cortical}\;\mathrm{effort}\right)\right]/\sqrt{2}$, where z represents standardized scores. Here, the outcome was described by variables such as response time, accuracy, number of correct responses, and attentional flexibility while mental effort was assessed based on fNIRS-measured HbO. Effort and outcome values were converted into Z-scored measurements and then efficiency was computed using the distance of the point from the zero-efficiency line (i.e., where unit performance score is equal to the effort score) and assessed as dependent measurements using statistical tests [39]; this approach was utilized in diverse neuroergonomics studies by da Silva Soares, Ramirez-Chavez [36,41,42,43].

DSST models for behavioral performance utilized the independent variables of participant mean response time, and the number of correct responses per block were compared for each segment of the task (*Task Block*) as well as for novice and experienced skill level (*Group*). Additionally, independent variables for HbO and reaction time-based neural efficiency (NeurEff_RxTime_) at every optode location (optodes 1–16) of the prefrontal cortex were compared for each segment of the task (*Task Block*) as well as for novice and experienced skill levels (*Group*). All fixed terms were held as both between and within fixed factors for comparisons.

For the single-search component of the DVST, independent variables of participant mean response time and accuracy were compared between the central search condition and peripheral search condition of the task (*Condition*) as well as for novice and experienced skill level (*Group*). For neural-based measures, independent variables for HbO and reaction time-based neural efficiency (NeurEff_RxTime_) at every optode location (optode 1–16) of the prefrontal cortex were compared between *Conditions* as well as for *Group*.

In terms of dual-search condition DVST measures, independent variables of participant mean response time, accuracy, and attentional delay (mean response times for peripheral search trials subtracted from mean response times for central search trials per task block) were compared between novice and experienced skill level (*Group*). Independent variables for HbO, reaction time-based neural efficiency (NeurEff_RxTime_), and attentional flexibility-based neural efficiency (NeurEff_Flexibilty_) were compared at every optode location (1–16) of the prefrontal cortex for *Group*.

The Stroop task’s independent variables of participant mean response time and accuracy were compared between the congruent and incongruent conditions of the task (*Condition*) as well as for novice and experienced skill level (*Group*). Independent variables for HbO and reaction time-based neural efficiency (NeurEff_RxTime_) at every optode location (optodes 1–16) of the prefrontal cortex were compared between the congruent and incongruent conditions of the task as well as for novice and experienced skill level (*Group*).

## 3. Results

### 3.1. DSST Results

#### 3.1.1. Response Time

A main effect was observed for *Task Block,* where the mean response times for all participants became significantly faster (F_2,347_ = 4.03, *p* < 0.05) as the task progressed (Block 1 = 0.9196 s, SEM = 0.0202; Block 2 = 0.8649 s, SEM = 0.0203; Block 3 = 0.8404 s, SEM = 0.0203). Additionally, there was a main effect present for *Group* (F_1,347_ = 24.73, *p* < 0.001), with *experienced* players demonstrating an overall faster mean response time (0.8168 s, SEM = 0.0177) compared to *novice* players (0.9331 s, SEM 0.0152).

#### 3.1.2. Number of Correct Responses

Over the duration of the DSST, there was a significant increase in participant scores (number of correct responses) with each repetition of the task block (F_2,347_ = 34.45, *p* < 0.05), with task scores improving from the first block to the last (Block 1 = 58.7103, SEM = 1.0069; Block 2 = 61.7575, SEM = 1.0132; Block 3 = 62.8110, SEM = 0.0132). Furthermore, there was a significant main effect for *Group* (F_1,347_ = 25.49, *p* < 0.001), with *experienced* players showing heightened response efficiency (64.04 correct, SEM = 0.89) over *novices* (58.15 correct, SEM = 0.76).

#### 3.1.3. fNIRS

While no main effects for *Group* were present at any optode, significant main effects for *Task Block* were identified for HbO signals at optode 5 (F_2,322_ = 6.33, *p* < 0.01), optode 7 (F_2,316_ = 5.57, *p* < 0.005), optode 8 (F_2,304_ = 4.18, *p* < 0.05), optode 9 (F_2,313_ = 7.00, *p* = 0.001), optode 10 (F_2,311_ = 4.12, *p* < 0.05), and optode 11 (F_2,300_ = 3.55, *p* < 0.05). For all optodes, hemodynamic activity increased monotonically from the start of the task to the end of the task.

#### 3.1.4. Neural Efficiency

**Figure 2:**
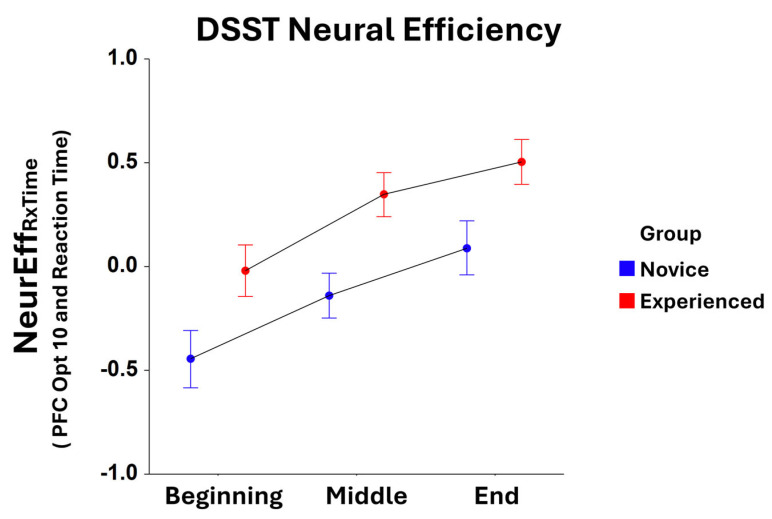
Comparison of reaction time-based NeurEff_RxTime_ at optode 10 between novice and experienced FPS players for each block of the DSST. There is a significant main effect (F_1,311_ = 19.07, *p* < 0.001) in the novice and experienced *Group* as well as a significant main effect (F_2,311_ = 9.27, *p* < 0.001) for *Task Block*. Whiskers are the standard error of the mean (SEM).

Significant main effects for the *Task Block* were found for NeurEff_RxTime_ at optode 1 (F_2,305_ = 3.65, *p* < 0.05), optode 3 (F_2,321_ = 7.40, *p* < 0.001), optode 4 (F_2,309_ = 6.42, *p* < 0.005), optode 5 (F_2,322_ = 11.58, *p* < 0.0001), optode 6 (F_2,308_ = 7.35, *p* < 0.001), optode 7 (F_2,316_ = 11.07, *p* < 0.001), optode 8 (F_2,304_ = 10.53, *p* < 0.001), optode 9 (F_2,313_ = 13.22, *p* < 0.001), optode 10 (F_2,311_ = 9.27, *p* < 0.001), optode 11 (F_2,300_ = 10.27, *p* < 0.001), optode 12 (F_2,304_ = 7.85, *p* < 0.001), optode 13 (F_2,309_ = 4.78, *p* < 0.01), optode 14 (F_2,310_ = 3.89, *p* < 0.05), and optode 15 (F_2,282_ = 4.03, *p* < 0.05). For all optodes, there were increases in NeurEff_RxTime_ from the initial task block to the final task block. Moreover, main effects for *Group* were observed for NeurEff_RxTime_ at optode 1 (F_1,305_ = 7.74, *p* < 0.01), optode 2 (F_1,300_ = 13.52, *p* < 0.001), optode 3 (F_1,321_ = 7.24, *p* < 0.01), optode 4 (F_1,309_ = 15.24, *p* < 0.001), optode 5 (F_1,322_ = 5.03, *p* < 0.05), optode 6 (F_1,308_ = 16.31, *p* < 0.001), optode 7 (F_1,316_ = 5.28, *p* < 0.05), optode 8 (F_1,304_ = 9.81, *p* < 0.005), optode 9 (F_1,313_ = 8.39, *p* < 0.005), optode 10 (F_1,311_ = 19.07, *p* < 0.001), optode 12 (F_1,304_ = 13.23, *p* < 0.001), and optode 14 (F_1,310_ = 6.24, *p* < 0.05). Here, neural efficiency was higher for experienced players compared to novices at every optode location, with optode 10 being most significant (Figure 2).

### 3.2. Single-Search DVST Results

#### 3.2.1. Response Time

Response times for all participants were first compared for the single-search conditions (*central search condition and peripheral search condition*) of the DVST. Here, there was a significant main effect for *Condition* as mean central search response times (0.8525 s, SEM = 0.0235) were significantly faster (F_1,234_ = 11.14, *p* < 0.001) than the mean peripheral search response times (0.9634 s, SEM = 0.0235). No main effect from *Group* was observed.

#### 3.2.2. Accuracy

Participants demonstrated significantly higher accuracy (F_1,234_ = 195.92, *p* < 0.001) in responses during the *peripheral search condition* (0.8792, SEM < 0.01) as compared to the *central search condition* (0.6884, SEM = 0.0096) of the DVST, with no main effect for *Group* present.

#### 3.2.3. fNIRS

Changes in HbO for participants during the *central search condition* and the *peripheral search condition* were compared. While no main effects for *Group* were present at any optode, significant main effects for *Condition* were identified for HbO signals at optode 6 (F_1,212_ = 4.58, *p* < 0.05), 14 (F_1,203_ = 6.04, *p* < 0.05) and 16 (F_1,186_ = 4.48, *p* < 0.05). For all optodes, the *central search condition* resulted in higher activity compared to the *peripheral search condition*. Next, significant *Group* main effects were observed for HbO signals in the *dual-search condition* alone at optode 2 (F_1,202_ = 5.44, *p* < 0.05), 3 (F_1,214_ = 5.82, *p* < 0.05), 4 (F_1,210_ = 6.54, *p* < 0.05), 5 (F_1,219_ = 4.28, *p* < 0.05), 6 (F_1,209_ = 5.76, *p* < 0.05), 7 (F_1,209_ = 4.60, *p* < 0.05), 8 (F_1,199_ = 5.03, *p* < 0.05), and 9 (F_1,211_ = 6.94, *p* < 0.01). For all optode locations, experienced players displayed higher HbO activity compared to novices in the *dual-search condition*.

#### 3.2.4. Neural Efficiency

There were no significant main effects found for NeurEff_RxTime_ for *Condition* or *Group* factors.

### 3.3. Dual-Search DVST Results

#### 3.3.1. Response Time

When comparing mean response times within the *dual-search condition* of the task alone, no main effect for *Group* was observed.

#### 3.3.2. Accuracy

When accuracy measures are compared for participants within the *dual-search condition* of the task alone, there is no main effect for *Group.*

#### 3.3.3. Attentional Delay

Within the *dual-search condition* of the DVST, the mean response times for peripheral search trials were subtracted from the mean response times for central search trials to obtain a metric for attentional delay between cognitive strategies for each participant. Here, there was a significant difference between *Groups* (F_1,117_ = 4.36, *p* < 0.05), with novices displaying increased delay (0.1093 s, SEM = 0.0501) compared to experienced players (0.0484 s, SEM = 0.0220).

#### 3.3.4. fNIRS

Significant *Group* main effects were observed for HbO signals in the *dual-search condition* alone at optode 2 (F_1,202_ = 5.44, *p* < 0.05), 3 (F_1,214_ = 5.82, *p* < 0.05), 4 (F_1,210_ = 6.54, *p* < 0.05), 5 (F_1,219_ = 4.28, *p* < 0.05), 6 (F_1,209_ = 5.76, *p* < 0.05), 7 (F_1,209_ = 4.60, *p* < 0.05), 8 (F_1,199_ = 5.03, *p* < 0.05), and 9 (F_1,211_ = 6.94, *p* < 0.01). For all optode locations, experienced players displayed higher HbO activity compared to novices.

#### 3.3.5. Neural Efficiency

**Figure 3:**
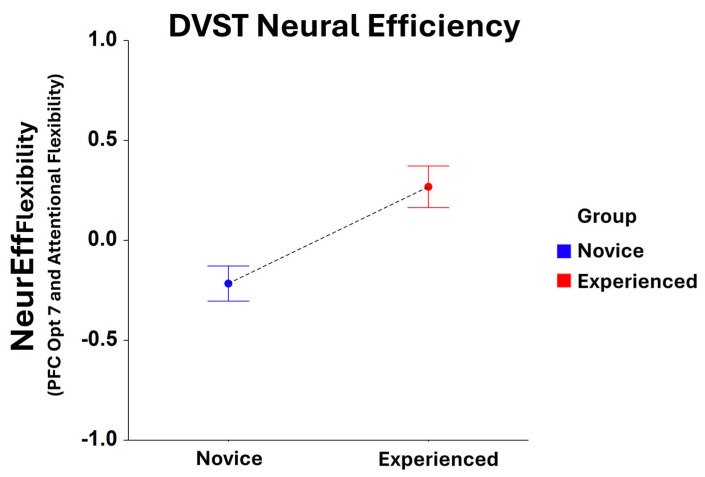
Comparison of NeurEff_Flexibility_ at optode 7 for novice and experienced FPS players performing the dual-search condition of the DVST. There is a significant main effect (F_1,209_ = 12.34, *p* = 0.001) in the novice and experienced *Groups*. Whiskers are SEM.

While no significant main effects were found for NeurEff_RxTime_ for *Group* in the *dual-search condition*, significant *Group* main effects were identified for NeurEff_Flexibilty_ at optode 1 (F_1,208_ = 6.39, *p* < 0.05), 2 (F_1,202_ = 8.43, *p* < 0.005), 3 (F_1,214_ = 10.91, *p* = 0.001), 4 (F_1,210_ = 10.94, *p* = 0.01), 5 (F_1,219_ = 9.22, *p* < 0.005), 6 (F_1,209_ = 10.14, *p* < 0.005), 7 (F_1,209_ = 12.34, *p* < 0.001), 8 (F_1,199_ = 9.15, *p* < 0.005), 9 (F_1,211_ = 11.90, *p* < 0.001), 10 (F_1,204_ = 6.78, *p* < 0.01), 12 (F_1,200_ = 4.92, *p* < 0.05), and 16 (F_1,184_ = 4.01, *p* < 0.05). Here, NeurEff_Flexibilty_ was optimized for experienced players compared to novices at every optode location, with optode 7 being the most significant location (Figure 3).

### 3.4. Stroop Task

#### 3.4.1. Response Time

A main effect was observed for *Condition,* where the mean response times for all participants were significantly faster (F_1,468_ = 37.54, *p* < 0.001) in the *congruent condition* of the task (0.8372 s, SEM = 0.01) compared to the *incongruent condition* of the task (0.9274 s, SEM = 0.01). Additionally, there is a main effect present for *Group* (F_1,468_ = 6.51, *p* = 0.01), with experienced players demonstrating overall faster response times (0.8635 s, SEM = 0.0111) compared to novice players (0.9011 s, SEM = 0.0097). However, post hoc comparison tests show that these significant *Group* effects are present due to response times in the *congruent condition* for each respective *Group* as both experienced players (F_1,468_ = 18.93, *p* < 0.001) and novices (F_1,468_ = 18.78, *p* < 0.001) logged faster reaction times in the *congruent condition* compared to the *incongruent condition* within their respective groups with experienced players maintaining faster responses than novices in the *congruent condition*.

#### 3.4.2. Accuracy

There is a main effect for present for *Condition* (F_1,468_ = 16.45, *p* < 0.001) where a significant difference in correct response accuracy is observed (F_1,468_ = 16.45, *p* < 0.001) between participant mean correct response rate in the *congruent condition* of the task (0.9845, SEM = 0.0038) and the *incongruent condition* of the task (0.9628, SEM < 0.005). No main effect is observed for *Group* in the model.

#### 3.4.3. fNIRS

Changes in HbO for participants during the *congruent condition and the incongruent condition* of the Stroop Task were compared. Significant main effects for *Condition* were identified for HbO signals at optodes 1 (F_1,401_ = 4.19, *p* < 0.05), 3 (F_1,396_ = 13.80, *p* < 0.001), 4 (F_1,392_ = 9.80, *p* < 0.005), 6 (F_1,375_ = 4.15, *p* < 0.05), and 13 (F_1,387_ = 5.62, *p* < 0.05). For all optodes, the *incongruent condition* resulted in higher activity compared to the *congruent condition*. Next, significant *Group* main effects were observed for HbO signals at optode 7 (F_1,398_ = 4.67, *p* < 0.05), 8 (F_1,368_ = 3.88, *p* < 0.05), and 10 (F_1,366_ = 9.44, *p* < 0.005). Here, *experienced* players displayed higher activity compared to novices at these optodes. More importantly, the interaction effect of *Group* * *Condition* was found to be significant at optode 10 (F_1,366_ = 4.25, *p* < 0.005) and 14 (F_1,380_ = 6.04, *p* = 0.01). Post hoc comparison tests for optode 10 with Bonferroni correction showed that within the *incongruent condition* of the task, experienced players demonstrated higher HbO activity than novices (F_1,366_ = 13.24, *p* < 0.001).

#### 3.4.4. Neural Efficiency

**Figure 4:**
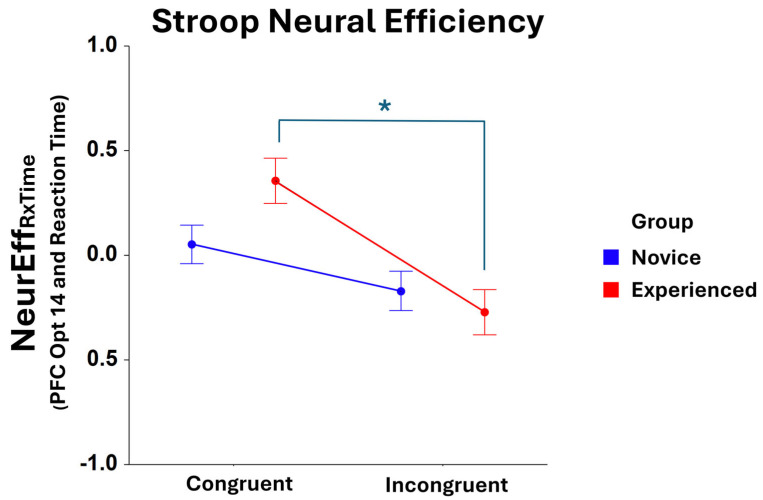
Comparison of NeurEff_RxTime_ at optode 14 for novice and experienced FPS players in the Stroop task. There is a significant main effect (F_1,209_ = 12.34, *p* < 0.001) between the congruent and incongruent *conditions* of the task as well as * a significant interaction for novice and experienced *Group* and *Condition* (F_1,380_ = 4.06, *p* < 0.05). A significant post hoc contrast is observed for the experienced group: congruent vs. incongruent (F_1,380_ = 17.51, *p* < 0.001). Whiskers are SEM.

Significant *Condition* main effects were found for NeurEff_RxTime_ at optode 1 (F_1,401_ = 25.64, *p* < 0.001), optode 2 (F_1,374_ = 22.34, *p* < 0.001), optode 3 (F_1,396_ = 37.18, *p* < 0.001), optode 4 (F_1,392_ = 35.37, *p* < 0.001), optode 5 (F_1,405_ = 21.23, *p* < 0.001), optode 6 (F_1,375_ = 25.50, *p* < 0.001), optode 7 (F_1,398_ = 19.87, *p* < 0.001), optode 8 (F_1,368_ = 17.55, *p* < 0.001), optode 9 (F_1,395_ = 16.58, *p* < 0.001), optode 10 (F_1,366_ = 14.29, *p* < 0.001), optode 11 (F_1,376_ = 20.28, *p* < 0.001), optode 12 (F_1,371_ = 20.75, *p* < 0.001), optode 13 (F_1,387_ = 24.00, *p* < 0.001), optode 14 (F_1,380_ = 17.78, *p* < 0.001), optode 15 (F_1,352_ = 18.52, *p* < 0.001), and optode 16 (F_1,336_ = 21.18, *p* < 0.001). For all optodes, NeurEff_RxTime_ was higher in the *congruent condition* compared to the *incongruent condition* of the Stroop Task. An interaction effect of *Group* * *Condition* was also found to be significant at optode 14 (F_1,380_ = 4.06, *p* < 0.05), with post hoc comparison tests showing that experienced players exhibit compromised neural efficiency in the incongruent condition compared to the congruent condition of the task (F_1,380_ = 17.51, *p* < 0.001), as shown in Figure 4. No other optode produced a significant NeurEff_RxTime_ result for *Group* * *Condition.*

## 4. Discussion

The present study aimed to explicate how prolonged FPS gaming experience influences the behavioral performance and neural efficiency of cognitive control (CC) processes. By employing three established cognitive tasks, DSST for *updating*, DVST for *attentional flexibility* or *shifting*, and the Stroop Task for *inhibition*, we examined how novice and experienced FPS gamers differ in behavioral outcomes and PFC hemodynamic activity, as measured by fNIRS wearable neuroimaging.

### 4.1. Updating and the DSST

Our DSST findings demonstrate that experienced FPS players responded both faster and more accurately than their novice counterparts. These data echo prior observations [23], indicating that well-practiced cognitive routines—such as rapid symbol–digit matching—may be reinforced by the fast-paced demands of FPS gameplay. Notably, while both groups exhibited a significant task-load effect in prefrontal oxyhemoglobin (HbO) levels over the duration of the DSST, no clear *between*-*group* difference in HbO emerged. This suggests that although both novices and experienced gamers increasingly recruited PFC resources as the task demands accumulated, the *relative* volume of PFC activation remained comparable across groups.

However, NeurEff_RxTime_ metrics reveal that experienced players employed these prefrontal resources more efficiently for each correct response. In other words, despite similar overall levels of HbO, experienced players achieved higher accuracy and faster responses, indicating that their FPS-based training may sharpen the ability to allocate cognitive resources selectively. These results add nuance to previous reports showing that trained individuals can achieve performance advantages without necessarily demonstrating a *greater* magnitude of PFC activation [3]. Instead, the critical distinction often lies in *how* these neural resources are utilized, a hallmark of “neural efficiency” in expert populations [4].

### 4.2. Attentional Flexibility and the DVST

The DVST was designed to parse out how gaming experience might influence shifting and search strategies under more complex conditions. While no significant group differences were found in single or dual-search accuracy, we did observe that experienced gamers switched more fluidly between central and peripheral targets during the dual-search component. This faster switching aligns with a body of work suggesting that intensive FPS gameplay trains players to handle multiple attention-demanding stimuli more rapidly [5,6].

From a neurophysiological standpoint, our fNIRS results support these behavioral observations and align with existing research [7], indicating that heightened CC performance under escalating cognitive demands is accompanied by increased PFC engagement. Critically, we observed not only an upregulation of HbO in the experienced group but also improved NeurEff_Flexibility_. This suggests that when faced with higher complexity, experienced gamers may recruit PFC networks both more robustly and more *effectively* to support rapid attentional shifting and the integration of competing visual information. These findings bolster the notion that repeated exposure to FPS gameplay refines the neural circuitry underlying flexible visual search and the timely updating of mental sets.

### 4.3. Inhibition and the Stroop Task

Consistent with expectations, participants’ performance declined in the incongruent Stroop condition relative to the congruent one, underscoring the classic interference effect associated with response inhibition demands [8]. Contrary to what might be seen in domains where strong inhibitory control is explicitly trained (e.g., certain sports or specialized tasks requiring high impulse control, like golf, archery, surgery, and piloting), our results revealed no significant behavioral advantage for experienced FPS gamers on incongruent trials. This aligns with earlier propositions that FPS environments may not actively encourage the *same* inhibitory skill development as they do for other CC subdomains like updating or shifting [9,10]. For instance, most causal first-person shooter games often lack penalties for “friendly fire”.

Interestingly, however, the experienced group did exhibit faster reaction times in the congruent Stroop condition. This faster baseline performance likely reflects the generalized quick-reflex advantage frequently reported among experienced FPS players [11]. From a neural standpoint, experienced players manifested significantly elevated medial prefrontal cortex (mPFC; optodes 7, 8, and 10) activity during incongruent trials compared to novices, suggesting a *greater* recruitment of executive control mechanisms to achieve *comparable* behavioral outcomes. While experienced gamers displayed superior or equal performance on incongruent trials in absolute terms, NeurEff_RxTime_ data revealed a significant interaction effect in the right lateral PFC (middle frontal gyrus) (optode 14). Specifically, experienced gamers showed substantially higher neural efficiency during congruent trials, compared to incongruent trials, showing a more pronounced Stroop effect at a neural-efficiency level. This significant contrast in efficiency between conditions was not observed to the same degree in novices. However, the neural efficiency of novices and experienced participants for incongruent trials were not significantly different. Thus, gaming experience did not appear to confer advantages for inhibition-depending trials (incongruent condition). Experienced players increased neural recruitment during inhibition-demanding trials, while achieving performance comparable to novices in the incongruent condition despite significant advantages during the congruent condition. This pattern suggests differential optimization for inhibitory control processes in experienced FPS players, potentially reflecting an adaptation to gaming environments where rapid responses may be rewarded over inhibitory control.

### 4.4. Implications

Taken together, these findings illustrate that prolonged FPS gameplay may bolster certain CC domains—particularly those related to rapid *updating* and *attentional shifting*—while providing less support for *response inhibition*. Although experienced gamers were able to mobilize the PFC more effectively in the DSST and DVST, they demonstrated a pattern of *increased* neural recruitment for the incongruent Stroop condition, but without a strong corresponding *behavioral* benefit. This pattern resonates with theories of cognitive plasticity that propose skill-specific adaptations in experts: repeated practice in certain tasks can yield considerable performance gains for those task-relevant processes, but such gains do not necessarily transfer to tasks that require a fundamentally different cognitive approach [12].

### 4.5. Limitations and Future Directions

Several avenues for future work as well as protocol limitations are worth highlighting. First, the current study did not differentiate between various FPS game types (e.g., tactical vs. run-and-gun) that may impose diverse inhibitory or attentional demands or attempt to compare the influence of other video game genres (e.g., real-time strategy, racing, simulators, puzzle games). Future research should include multi-genre gaming comparisons to isolate FPS-specific effects from the general effects of gaming experience.

Second, although video game experience is increasingly common amongst the population at large, the study population was primarily young-adult and experienced FPS gamers were predominantly male. In addition, binary classification of experienced gamers may overlook dose-dependent relationships between gaming and cognition, potentially revealing more nuanced relationships between practice intensity and cognitive control development. Other potential confounders may include participant fatigue and time-of-day variability in testing, as well as other individual differences which may affect both behavioral performance and neural measurements.

Third, while fNIRS offers high ecological validity and moderate spatial resolution in measuring cortical oxygenation, combining it with complementary methods (e.g., electroencephalography, EEG, for temporal resolution or functional magnetic resonance imaging, fMRI, for deeper subcortical insights) would provide a more comprehensive mapping of neural processes.

Lastly, although we noted that the experienced group performed better in tasks emphasizing updating and shifting, a longitudinal study design would be ideal to confirm a direct causal link between FPS training and changes in CC and PFC activation patterns. However, even with longitudinal examination, continued gaming practice which helps develop eventual expertise may also involve a form of performance-based self-selection in which individuals who show early advantages in a particular activity are also more likely to continue practice, dramatizing “natural” differences between experience and novices [44].

## 5. Conclusions

Overall, our findings make a strong case that the intense cognitive demands of FPS games foster improvements in updating and shifting processes, reflected by both faster behavioral performance and more efficient PFC resource allocation. In contrast, the inhibitory control domain appears less influenced—or even detrimentally shaped—by prolonged FPS experience, as evidenced by the increased mPFC activation required for incongruent Stroop trials in experienced gamers. These discoveries not only refine our understanding of how specific dimensions of CC respond to domain-specific training but also open new perspectives on leveraging fNIRS in skill acquisition research. By mapping the neural signatures of CC development in real-world contexts, this work contributes to a broader neuroergonomic framework that can be extended to other high-performance domains requiring rapid decision making and attentional agility.

## Data Availability

The original contributions presented in the study are included in the article/supplementary material; further inquiries can be directed to the corresponding author.

## Supplementary Materials

The following supporting information can be downloaded at https://www.mdpi.com/article/10.3390/brainsci15060568/s1, Table S1: Demographic characteristics and gaming experience of participants (N = 120); Table S2: Cognitive performance measures comparing novice and experienced participants.
