# Supplementary material for: Cognitive Control and Prefrontal Neural Efficiency in Experienced and Novice E-Gamers
Source: Brain Sci. 2025 May 25;15(6):568. doi: 10.3390/brainsci15060568 (PMC12191396; doi:10.3390/brainsci15060568)
Supplement: Supplementary file 1 [file brainsci-15-00568-s001.zip › brainsci-3611338-supplementary.pdf]

Supplementary Material

for

Cognitive Control and Prefrontal Neural Efficiency in Experienced and Novice E-Gamers

Jan Watson [1], Adrian Curtin [1], Yigit Topoglu [1], Rajneesh Suri [2,3], Hasan Ayaz [1,3,4,5,6*]

[1] School of Biomedical Engineering. Science and Health Systems, Drexel University, Philadelphia, PA 19104, USA
[2] Lebow College of Business, Drexel University, Philadelphia, PA 19104, USA
[3] Drexel Solutions Institute, Drexel University, Philadelphia, PA 19104, USA
[4] Department of Psychological and Brain Sciences, College of Arts and Sciences, Drexel University, Philadelphia, PA 19104, USA
[5] A.J. Drexel Autism Institute, Drexel University, Philadelphia, PA 19104, USA
[6] Center for Injury Research and Prevention, Children's Hospital of Philadelphia, Philadelphia, PA 19104, USA

Table S1. Demographic characteristics and gaming experience of participants (N = 120).

Measure	Total Cohort	Novice	Expert	Statistical Analysis
Gender (Male/Female)	90/30	43/27	47/3	$\chi^2 = 16.50$, df = 1, $p < 0.001$ ***
Age (years)	21.7 ± 3.8	22.5 ± 4.2	20.6 ± 2.6	$t(118) = -2.89$, $p = 0.005$ **
Gaming frequency (hours/week)	10.2 ± 9.0	6.4 ± 6.6	15.5 ± 9.3	$t(118) = 6.22$, $p < 0.001$ ***

Note: Values are presented as mean ± SD unless otherwise specified.
Statistical significance: ** $p < 0.01$, *** $p < 0.001$.

Table 2. Cognitive performance measures comparing novice and experienced participants.

Measure	Novice by Gender			Experienced by Gender			Overall		Group
	Male	Female	P-Value	Male	Female	P-Value	Novice	Experienced	P-Value
**DSST Measures**									
Response Time (RT)	0.97 s	0.86 s	$F_{1,201}$=7.21, p<0.01	0.81 s	0.77 s	ns	0.93 s (0.02)	0.82 s (0.02)	$F_{1,347}$=24.73, p<0.001
Correct Response	0.97 (0.00)	0.97 (0.00)	ns	0.96 (0.00)	0.97 (0.01)	ns	58.15 (0.76)	64.04 (0.89)	$F_{1,347}$=25.49, p<0.001
**DVST Measures**									
Single Search Central RT	0.89 s (0.03)	0.81 s (0.05)	ns	0.84 s (0.04)	0.86 s (0.15)	ns	0.87 s (0.03)	0.84 s (0.03)	ns
Single Search Peripheral RT	0.97 s (0.04)	0.93 s (0.06)	ns	0.96 s (0.04)	1.11 s (0.16)	ns	0.96 s (0.03)	0.97 s (0.04)	ns
Single Search Central Accuracy	0.67 (0.01)	0.71 (0.02)	ns	0.70 (0.01)	0.72 (0.06)	ns	0.78 (0.01)	0.79 (0.01)	ns
Single Search Peripheral Accuracy	0.86 (0.02)	0.92 (0.02)	$F_{1,66}$=4.51, p<0.05	0.88 (0.02)	0.94 (0.06)	ns	0.88 (0.01)	0.88 (0.02)	ns
Dual Search RT	1.32 s (0.04)	1.36 s (0.05)	ns	1.34 s (0.03)	1.22 s (0.14)	ns	1.33 s (0.03)	1.34 s (0.03)	ns
Dual Search Accuracy	0.69 (0.01)	0.72 (0.01)	ns	0.72 (0.01)	0.74 (0.04)	ns	0.70 (0.01)	0.72 (0.01)	ns
**Stroop Measures**									
Congruent RT	0.88 s (0.14)	0.82 s (0.02)	$F_{1,132}$=3.97, p=0.05	0.82 s (0.02)	0.73 s (0.07)	ns	0.86 s (0.01)	0.82 s (0.02)	$F_{1,234}$=4.63, p<0.05
Incongruent RT	0.97 s (0.02)	0.89 s (0.03)	$F_{1,132}$=5.78, p<0.05	0.92 s (0.02)	0.80 s (0.06)	ns	0.94 s (0.01)	0.91 s (0.02)	ns
Congruent Accuracy	0.99 (0.00)	0.98 (0.01)	ns	0.98 (0.00)	0.97 (0.02)	ns	0.99 (0.00)	0.98 (0.00)	ns
Incongruent Accuracy	0.96 (0.01)	0.98 (0.01)	$F_{1,132}$=4.70, p<0.05	0.96 (0.01)	0.99 (0.03)	ns	0.96 (0.01)	0.96 (0.01)	ns

Note: Values are presented as mean (standard error) unless otherwise specified. DSST = Digit Symbol Substitution Test; DVST = Digit Vigilance Search Task; RT = Response Time; ns = not significant (p > 0.05). Subscript numbers in F statistics represent degrees of freedom.
